# Genome-wide Identification and Characterization of the GRAS Transcription Factors in Garlic (*Allium sativum* L.)

**DOI:** 10.3389/fpls.2022.890052

**Published:** 2022-04-12

**Authors:** Xueyu Zhang, Xiai Yang, Qiaoyun He, Yanzhou Wang, Guolu Liang, Touming Liu

**Affiliations:** ^1^Institute of Bast Fiber Crops, Chinese Academy of Agricultural Sciences, Changsha, China; ^2^College of Horticulture and Landscape Architecture, Southwest University, Chongqing, China

**Keywords:** *Allium sativum*, GRAS gene family, GA3 treatment, lighting response, DELLA

## Abstract

GRAS transcription factors play crucial roles in plant growth and development and have been widely explored in many plant species. Garlic (*Allium sativum* L.) is an important crop owing to its edible and medicinal properties. However, no GRAS transcription factors have been identified in this crop. In this study, 46 garlic GRAS genes were identified and assigned to 16 subfamilies using the GRAS members of *Arabidopsis thaliana*, *Oryza sativa*, and *Amborella trichopoda* as reference queries. Expression analysis revealed that garlic GRAS genes showed distinct differences in various garlic tissues, as well as during different growth stages of the bulbs. Five of these 46 genes were identified as DELLA-like protein-encoding genes and three of which, *Asa2G00237.1*/*Asa2G00240.1* and *Asa4G02090.1*, responded to exogenous GA3 treatment, and showed a significant association between their transcription abundance and bulb traits in 102 garlic accessions, thereby indicating their role in regulating the growth of garlic bulbs. These results will lay a useful foundation for further investigation of the biological functions of GRAS genes and guiding the genetic breeding of garlic in the future.

## Introduction

The expression of most eukaryotic genes relies on the specific transcription factors (TFs) that bind to or modulate the DNA structure in the regulatory region of genes by activating RNA polymerase to initiate transcription. One of the most important families of transcription factors in plants is the GRAS family, which is named after its first three identified members: gibberellic acid intensive (*GAI*), repressor of GAI-3 mutant (*RGA*), and Scarecrow (*SCR*). GRAS proteins are typically composed of 400–700 amino acid residues with a variable N-terminal structure. They contain five critical and conserved domains: leucine-heptad repeat I (LHR I), Val-His-Ile-Ile-Asp (VHIID), leucine-heptad repeat II (LHR II), Pro-Phe-Tyr-Arg-Glu (PFYRE), and Ser-Ala-Trp (SAW; Pysh et al., 1999). The representatives of dicotyledons and monocotyledons, *Arabidopsis thaliana* and rice contain 33 and 57 *GRAS* members, respectively; of these, eight GRAS subfamilies, namely, LISCL, PAT1, SCL3, DELLA, SCR, SHR, LS, and HAM, are common in both plants (Tian et al., 2004). Recently, more GRAS members consisting of 17 subfamilies have been identified in eight species (Cenci and Rouard, 2017). A total of 9,304 GRAS genes have been added to the Plant transcription factor database (PlantTFDB) so far (Jin et al., 2017), indicating that this family is one of the largest gene families.

GRAS TFs are multifunctional proteins that play various roles in plant growth and development, including gibberellin and (Sun and Gubler, 2004) phytochrome A signal transduction (Bolle et al., 2000), axillary meristem initiation (Tanaka et al., 2015), shoot meristem maintenance (Jha et al., 2020), and root radial patterning (Sabatini et al., 2003). Members of the LS subfamily mainly function as regulators of bud outgrowth; for example, rice *MONOCULM 1* (*MOC1*) is essential for forming axillary meristems and controlling tiller number (Schumacher et al., 1999; Greb et al., 2003). DELLA-like proteins are another type of GRAS TFs with conserved domains of DELLA and TVHYNP and act as repressors of GA signaling, thereby regulating plant growth, including stem elongation (Sun and Gubler, 2004), secondary cell wall biosynthesis (Huang et al., 2015), and stress response (Maggio et al., 2010). DELLA proteins also regulate the MOC1 degradation in rice to control tiller number (Liao et al., 2019). In addition, members of the HAM subfamily are involved in the WUS-CLV3 interaction module, which coordinates with auxin and cytokinin to control the maintenance of stem cells and the differentiation of shoot apical meristems (SAM; Zhou et al., 2015, 2018). In Arabidopsis, *SCR* and *SHR* regulate the radial patterning of roots (Di Laurenzio et al., 1996; Helariutta et al., 2000), whereas *PAT1* responds to light signaling (Bolle et al., 2000).

Being cultivated for over 5,000 years, Garlic (*A. sativum*) is not only widely consumed as green nutritional vegetable but also used effectively in medicinal and nutraceutical industries (Martin and Ernst, 2003; Kamenetsky et al., 2015). Bulbs are the main consumed organ of garlic and consist of several cloves, which are abnormal buds that undergo enlarged growth. GRAS members are known to play crucial roles in regulating axillary meristem initiation and bud outgrowth (Sun et al., 2012). Furthermore, a recent study indicated the potential association between genes involved in SAM development and garlic bulb growth (Sun et al., 2020). Therefore, GRAS members probably play a role in the bulb growth of garlic and identification and characterization of GRAS members will be helpful for further research on bulb growth in this *Allium* crop. However, none of the GRAS members have been identified and reported in garlic. In the present study, we conducted a systematic investigation of GRAS members in garlic and the results provide a basis for analyzing their function in the future.

## Materials and Methods

### Identifying *GRAS* Genes

The GRAS protein sequences in *Arabidopsis* were downloaded from the TAIR database^1^ (Tian et al., 2004) and those in rice and *Amborella trichopoda* were collected and published by Alberto et al. (Cenci and Rouard, 2017). The GRAS proteins of the three plant species were used as reference queries to identify GRAS proteins in garlic using BLASTP (Chen et al., 2020). Subsequently, the preliminarily identified proteins were subjected to conserved domain region analysis using the CDD program in NCBI^2^ (Lu et al., 2020) and MEME^3^ (Bailey et al., 2009). After manual verification, only proteins with lengths greater than 200 amino acids and specific GRAS domains were selected for subsequent analysis. The basic features of GRAS proteins, including molecular weight (MW), coding sequence length (CDS), and isoelectric point (pI), were predicted using the ExPASy software^4^ (Gasteiger et al., 2005), and subcellular localizations were predicted by Localizer 1.0.4 (Sperschneider et al., 2017).

### Chromosomal Location, Conserved Motifs, and Gene Structures Analysis

The annotation of garlic genome (Sun et al., 2020), locastion of *GRAS* genes on the chromosomes/scaffolds was visualized using TBtools (v1.098696; Chen et al., 2020). Multiple sequence alignments of all GRAS proteins were conducted using the JalView (version 2.10.3) software to investigate the protein domains (Waterhouse et al., 2009). The conserved motifs were analyzed using two online tools: MEME (Bailey et al., 2009) and batch CD-Search (Lu et al., 2020). Protein structures and motifs were visualized using TBTools (Chen et al., 2020).

### Expression Analysis and Interaction Network of *GRAS* Genes

Tissue-specific expression pattern of *AsGRAS* genes was analyzed by extracting the expression data from the reported transcriptome analysis of garlic (Sun et al., 2020) and an expression heatmap was obtained using TBtools (Chen et al., 2020). Zhu et al. (2019) completed the mRNA sequencing of enlarged bulbs from 102 accessions and introduced an associated transcriptomic method to identify genes related to transcript abundance and bulb traits (Zhu et al., 2019). Based on the expression data of these 102 accessions and the associated transcriptomic method, we performed a correlation analysis of bulb traits and transcript abundance of garlic DELLA genes. Orthologous gene pairs between *AsGRAS*s and *AtGRAS*s were identified and the interaction network of orthologous *GRAS* genes was predicted using the STRING 11.5 database (von Mering et al., 2005).

### GA3 Treatment and *DELLA* Expression

Approximately 100 garlic cloves (cv. Ershuizao) were planted in a pot and grown in a greenhouse with a 12 h light period. After 1 month, half of the garlic seedlings were treated by spraying exogenous GA3 and the other half served as the control group. Briefly, GA3 (200 mg/l) and sterile distilled water were sprayed on the leaves of treatment and control groups, respectively. Leaf samples were collected in triplicates from both groups after 0, 4, and 16 h of treatment and were pre-ground to a powder in liquid nitrogen. Total RNA extraction was performed using SteadyPure plant RNA extraction kit AG21019 [Accurate Biotechnology (Hunan) Co., Ltd.] and resultant RNA population was qualified by gel electrophoresis followed by first-strand cDNA biosynthesis [Evo M-MLV RT premix for qPCR AG11706, Accurate Biotechnology (Hunan) Co., Ltd.]. qRT-PCR analysis was performed using CFX96 Real-Time PCR Detection System (Bio-Rad Laboratories, United States) with SYBR green Premix Pro Taq HS qPCR Kit AG11701 [Accurate Biotechnology (Hunan) Co., Ltd.]. Primer sequences are listed in Supplementary Table S1.

## Results

### Identification of *GRAS* Genes in Garlic

**Figure 1:**
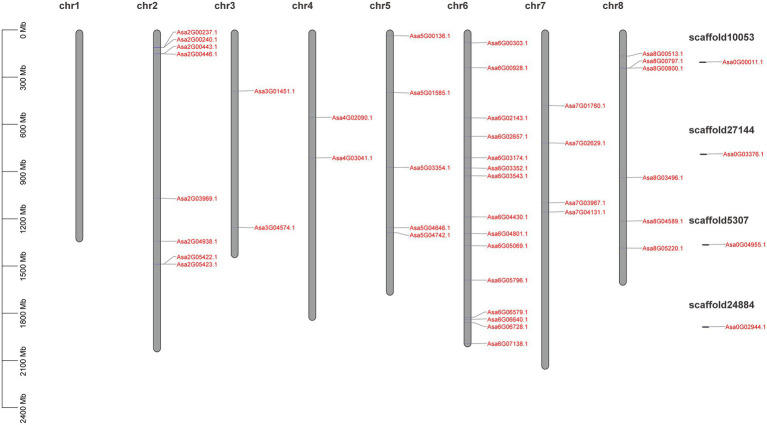
Schematic representation of the chromosomal distribution of the *AsGRAS* genes. Vertical bars represent garlic chromosomes. The chromosome number is mentioned above each chromosome. The scale on the left represents chromosome length (Mb).

A total of 61 GRAS candidate genes were obtained from the garlic genome according to the BLAST results. Of these, 15 encoded a protein with <200 amino acids residues and/or without GRAS domains and were filtered. Finally, 46 GRAS members were identified which typically comprised of five conserved domains: LHRI, VHIID, LHRII, PFYRE, and SAW (Supplementary Figure S1; Supplementary Table S2). The 46 identified GRAS genes were distributed on seven of eight garlic chromosomes (except for chromosome 1) and their distribution varied among chromosomes, ranging from 2 to 15 genes per chromosome (Figure 1).

### Characterization of AsGRAS Members

The length of the putative GRAS proteins ranged from 202 to 705 amino acids and the predicted molecular weights ranged from 22 to 80 kDa (Table 1). The theoretical pI varied from 4.87 to 9.76. Except for *Asa4G03041.1*, all the proteins had a hydropathicity value of <0, indicating the hydrophilic nature of these GRAS proteins. Additionally, only seven proteins showed an instability index value of <40.0 (i.e., threshold value; Table 1), suggesting that most of the garlic GRAS proteins probably had an unstable structure. Subcellular localization prediction identified between 16 and 2 GRAS proteins located in the nucleus and chloroplast, respectively (Table 1). Interestingly, the *Asa3G01451.1* protein was predicted with position signals in both the nucleus and chloroplast.

**Table 1:** List of gene IDs and characteristics of the 46 *AsGRAS* genes.

No.	Name	Groups	Length (aa)	MW (Da)^a^	pI^b^	Instability index	GRAVY^c^	GRAS domain start	GRAS domain end	Subcellular location prediction
1	*Asa4G02090.1*	DELLA	513	56219.57	6.92	53.93	−0.351	147	508	Nucleus
2	*Asa2G00240.1*	DELLA	570	62413.46	5.08	47.15	−0.252	201	565	Nucleus
3	*Asa2G00237.1*	DELLA	570	62413.46	5.08	47.15	−0.252	201	565	Nucleus
4	*Asa6G07138.1*	DELLA	524	57489.78	5.31	50.77	−0.139	127	485	–
5	*Asa6G05069.1*	DELLA	393	42791.29	6	55.93	−0.177	127	332	–
6	*Asa6G03174.1*	DLT	425	49025.37	4.87	58.04	−0.386	61	421	–
7	*Asa5G03354.1*	DLT	394	45213.69	6.4	44.52	−0.343	62	391	–
8	*Asa0G02944.1*	HAM	570	63884.08	5.53	50.82	−0.253	219	569	–
9	*Asa2G05423.1*	HAM	589	65112.39	5.6	49.05	−0.16	238	589	Nucleus
10	*Asa2G05422.1*	HAM	589	65086.3	5.6	49.98	−0.169	238	589	Nucleus
11	*Asa6G02657.1*	HAM	598	66541.08	5.41	44.16	−0.154	242	598	–
12	*Asa7G03967.1*	HAM	598	66541.08	5.41	44.16	−0.154	242	598	–
13	*Asa6G04801.1*	HAM	469	51125.93	5.38	46.86	−0.01	104	468	–
14	*Asa8G05220.1*	LISCL	697	80098.07	5.6	49.42	−0.582	329	694	Nucleus
15	*Asa7G02629.1*	LISCL	704	79386.59	6.83	44.99	−0.459	338	702	–
16	*Asa0G04955.1*	LISCL	630	72705.89	6.01	48.41	−0.642	262	628	Nucleus
17	*Asa3G04574.1*	LISCL	249	29052.11	8.46	57.54	−0.616	1	244	–
18	*Asa7G04131.1*	LISCL	313	36431.77	8.25	39.36	−0.37	1	311	–
19	*Asa0G00011.1*	LISCL	445	49614.04	5.48	42.12	−0.541	290	432	Nucleus
20	*Asa5G04742.1*	LS	370	41594.31	6.36	50.39	−0.113	21	369	–
21	*Asa2G04938.1*	NSP2	507	56223.08	5.77	34.63	−0.398	119	489	–
22	*Asa2G03969.1*	PAT	507	57422.1	6.82	49.46	−0.551	143	506	Nucleus
23	*Asa0G03376.1*	PAT	507	57435.1	6.82	49.25	−0.557	143	506	Nucleus
24	*Asa6G03352.1*	PAT	521	57562.51	5.89	49.6	−0.198	154	521	–
25	*Asa7G01760.1*	PAT	490	54934.4	4.98	40.56	−0.239	126	490	Nucleus
26	*Asa4G03041.1*	RAD	383	42288.8	7.06	53.85	0.044	18	382	–
27	*Asa2G00446.1*	RAD	479	53865.06	5.41	47.84	−0.223	106	471	Nucleus
28	*Asa6G00928.1*	RAM1	699	77313.18	5.51	56.52	−0.314	347	698	–
29	*Asa6G04430.1*	RAM1	650	73009.45	5.57	64.6	−0.296	300	649	–
30	*Asa6G06640.1*	RAM1	650	73037.51	5.63	64.6	−0.297	300	649	–
31	*Asa8G00513.1*	SCL3	474	53343.2	6.17	52.57	−0.162	55	469	–
32	*Asa8G03496.1*	SCL3	472	53097.31	5.96	59.69	−0.24	58	464	–
33	*Asa6G00303.1*	SCL32	414	46449.05	5.34	39.58	−0.193	54	407	–
34	*Asa6G06579.1*	SCL32	414	46214.89	5.53	51.6	−0.062	49	411	–
35	*Asa6G06728.1*	SCL4/7	473	53333.23	5.07	47.1	−0.251	113	472	–
36	*Asa8G04589.1*	SCLA	453	51116.97	6.18	33.17	−0.365	84	449	Nucleus
37	*Asa6G05796.1*	SCLB	531	60030.24	5.43	38.91	−0.231	154	519	–
38	*Asa8G00797.1*	SCLB	563	63684.33	5.27	37.51	−0.235	186	551	–
39	*Asa8G00800.1*	SCLB	517	58563.02	5.92	33.15	−0.165	146	512	–
40	*Asa6G02143.1*	SCLB	484	55394.73	5.56	43.89	−0.1	116	477	–
41	*Asa3G01451.1*	SCR	616	67532.36	5.93	54.82	−0.277	251	601	Nucleus and Chloroplast
42	*Asa2G00443.1*	SCR	430	47240.07	5.48	45.16	−0.05	56	414	Nucleus
43	*Asa5G00136.1*	SCR	469	52390.33	5.94	47.13	−0.245	114	464	Nucleus
44	*Asa5G01585.1*	SCR	201	22372.98	9.76	41.23	−0.138	33	194	Chloroplast
45	*Asa6G03543.1*	SHR	480	54159.49	5.82	53.59	−0.478	109	479	–
46	*Asa5G04646.1*	SHR	428	48196.74	5.87	42.35	−0.251	60	427	–

a *Molecular weight*.

b *Isoelectric point*.

c *Grand average of hydropathy*.

**Figure 2:**
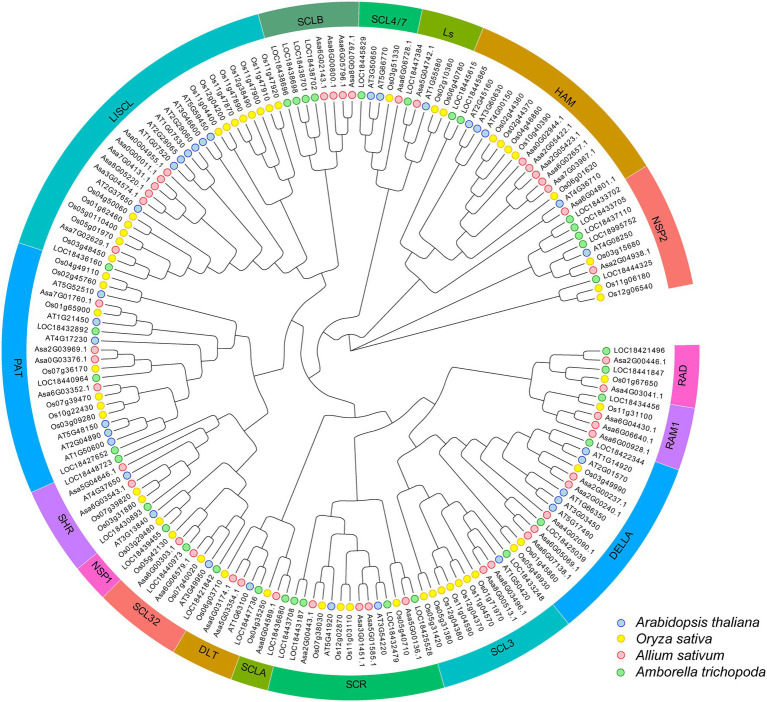
Unrooted phylogenetic tree representing relationships among GRAS families of four plant species. The phylogenetic tree was constructed using the NJ method and shows 17 subfamilies. GRAS proteins from *Allium sativum* L., *Arabidopsis*, *Oryza sativa,* and *Amborella trichopoda* are marked with red, blue, yellow, and green, respectively.

**Figure 3:**
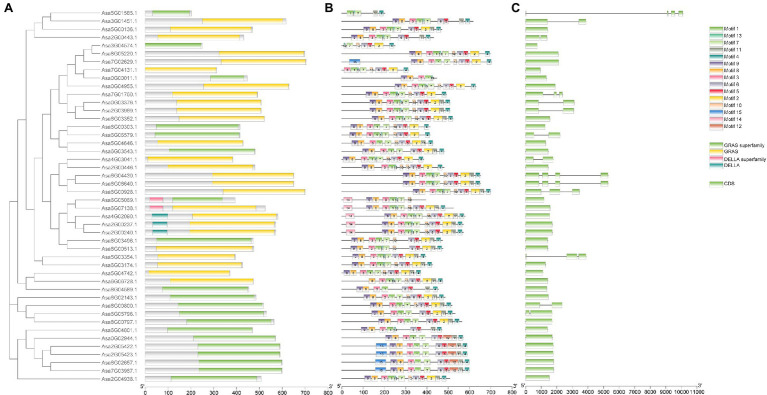
Phylogenetic relationship, gene structure analysis, and motif distribution of garlic *GRAS* genes. **(A)** Conserved domain structures of 46 *AsGRASs*. **(B)** Amino acid motifs in the *AsGRAS* proteins are represented by colored boxes. The black lines indicate relative protein lengths. **(C)** Exons and introns are indicated by rectangles and gray lines, respectively.

Phylogenetic analysis was performed for 46 AsGRAS members, together with 123 known GRAS proteins from *Arabidopsis* (33 members), *O. sativa* (56 members), and *A. trichopoda* (34 members). The results indicated that 46 AsGRAS members were assigned to 16 of the 17 known GRAS subfamilies (Figure 2). The protein structure investigation indicated that 46 AsGRAS proteins had conserved domains of the GRAS or GRAS superfamily, comprising of a conserved motif structure with LHRI-VHIID-LHRII-PRYRE-SAW domains (Table 1; Figure 3A). However, protein motifs showed larger differences between members from different subfamilies than from the same subfamily, suggesting a diversification of motif sequences among the garlic GRAS subfamily (Figure 3B; Supplementary Figure S2). The details of structural organization revealed that 32 of 46 *AsGRAS* genes had no introns; in particular, all genes of the LISCL, DELLA, SCL3, SHR, and HAM subfamilies lacked any introns, whereas all genes of the PAT1, RAM1, and SCR subfamilies had more than two exons (Figure 3C).

### Expression Pattern of *AsGRAS* Genes

**Figure 4:**
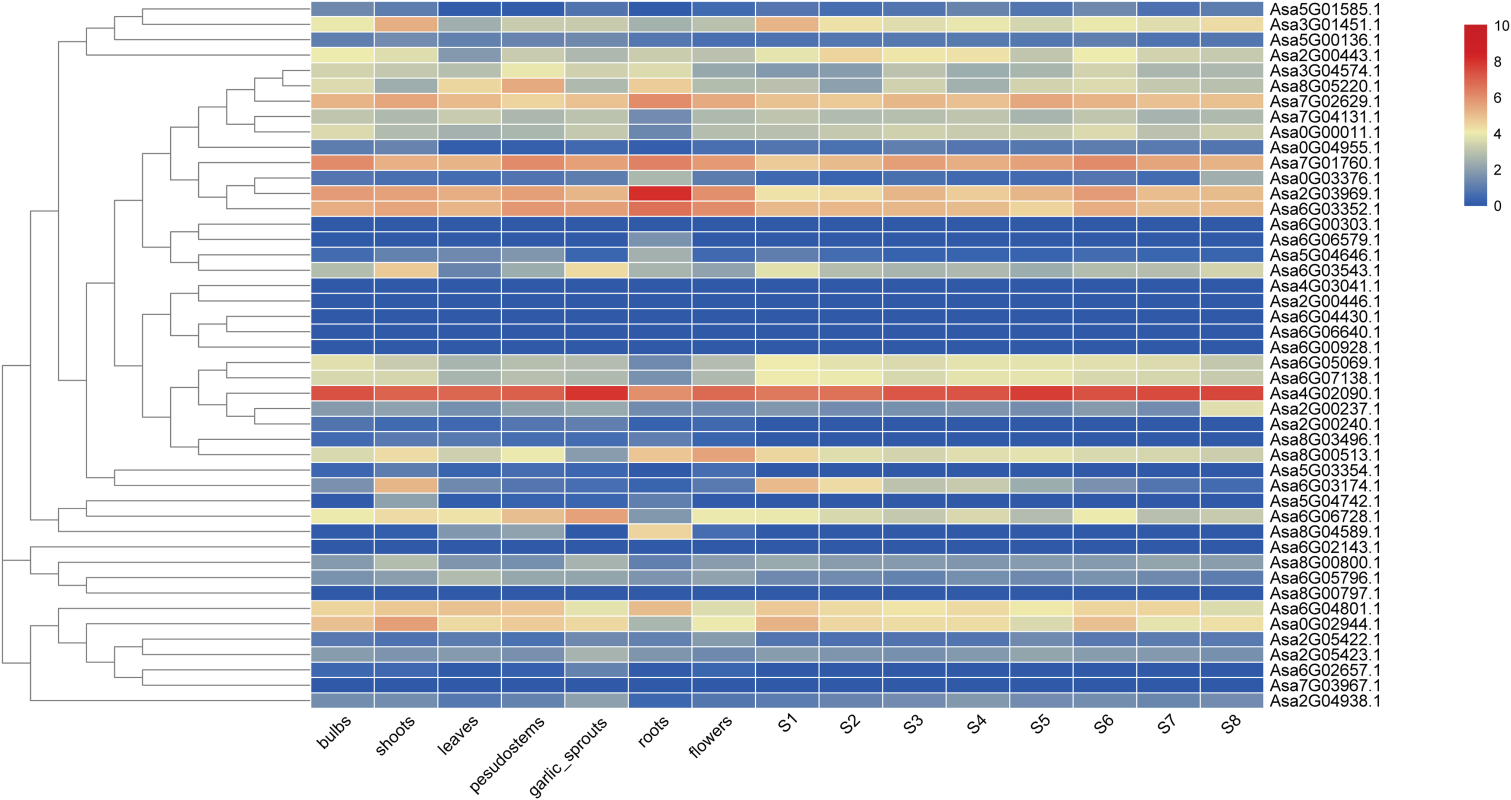
Expression heatmap of garlic *GRAS* genes in seven tissues and eight bulb growth stages. The colored scale on the right represents the degree of expression, which increases from blue to red.

The expression patterns of 46 *AsGRAS* genes in seven tissues and eight bulb-developmental stages were investigated and revealed significant differences (Figure 4). Notably, DELLA-like *Asa4G02090.1* showed the highest expression level in all the tissues except roots, whereas nine *AsGRAS* genes were not expressed at all in the 15 samples including, *Asa6G00928.1*, *Asa4G03041.1*, *Asa6G04430.1*, *Asa6G06640.1*, *Asa2G00446.1*, *Asa8G00797.1*, *Asa6G02143.1*, *Asa6G00303.1*, and *Asa7G03967.1*.

**Figure 5:**
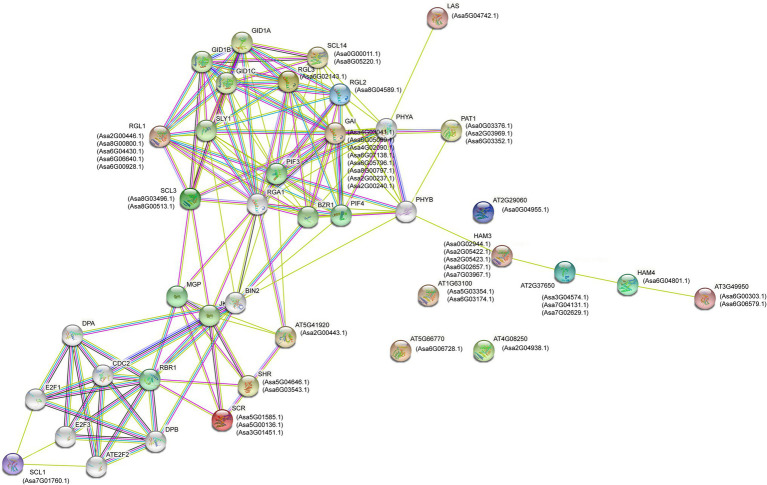
Protein–protein interaction network of the GRAS proteins in garlic with the orthologous ones in *Arabidopsis*.

To understand the biological function of AsGRAS TFs further, their protein–protein interactions were predicted using the ortholog-based method. The results showed that 46 AsGRAS members were orthologs of 20 Arabidopsis GRAS proteins that constituted an interacting network (Figure 5). According to the prediction, *AsGRAS* proteins interacted with PIF, BZR1, BIN2, and GID1 proteins, which are involved in GA and brassinolide signal transduction mechanism, and lighting-responsive PHYA and PHYB proteins. Furthermore, some members of SCL subfamilies showed a putative interaction with cell cycle regulatory DPA and E2F proteins, indicating a potential role of these garlic GRAS proteins in the regulation of cell cycle.

### DELLA Subfamily

As a subfamily of the AsGRAS family, the DELLA subfamily has been frequently studied as these proteins are the central repressors of gibberellin (GA) response (Park et al., 2013). Bioinformatics prediction identified five garlic DELLA members as *Asa2G00237.1*, *Asa2G00240.1*, *Asa4G02090.1*, *Asa6G05069.1*, and *Asa6G07138.1*. Of these DELLA-like genes, *Asa2G00237.1* and *Asa2G00240.1* showed a tandem repeat distribution in the genomic regions, with a complete identical coding sequence; whereas *Asa6G05069.1* and *Asa6G07138.1* displayed almost identical sequences in their coding regions.

**Figure 6:**
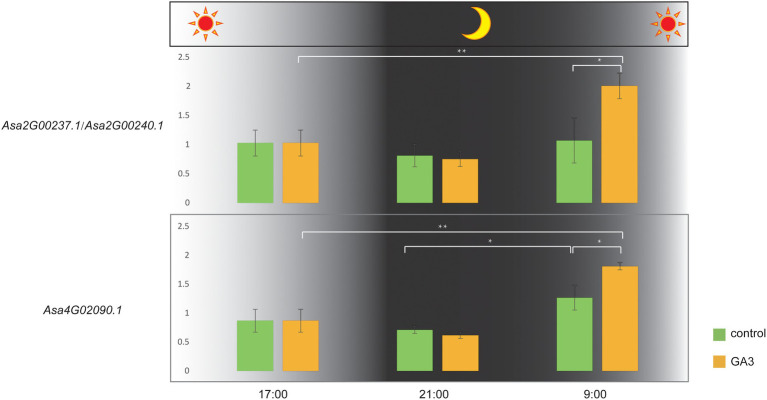
Expression differences of DELLA subfamily genes in one-month-old garlic seedlings sprayed with GA3 solution (sampling times: 0, 4, and 16 h after treatment). * and ** indicate significant differences at the 0.05 and 0.01 levels, respectively. Control: the same amount of distilled water treatment, GA3: 200 mg/l GA3 treatment.

**Figure 7:**
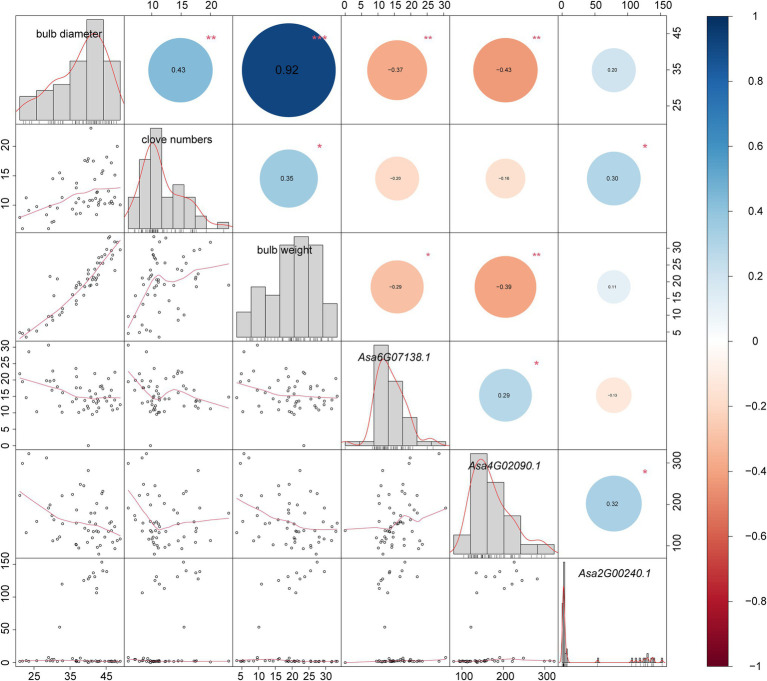
Correlation diagrams between garlic bulb traits (bulb diameter, bulb weight, and clove numbers) and *DELLA* genes. The correlation diagrams are represented by bar graphs, red curves, and scatter plots simultaneously. Numbers in colored dots are correlation coefficients, dot size indicates correlation degree, red indicates negative correlation, and blue indicates positive correlation. *, **, and *** indicate significant correlations at the 0.05, 0.01, and 0.001 levels, respectively. As the CDS sequences are identical, a pair of tandem repeat genes are represented by one gene ID.

The bulb is the main consumed organ of garlic and consists of several cloves. GA3 treatment can increase the number of cloves in garlic (Liu et al., 2019). In this study, we investigated the expression response of DELLA-like genes to GA3 treatment. Because a complete identical coding sequence makes a challenge to distinguish their transcripts, we performed the expression analysis for these two genes as a whole. Consequently, we found that *Asa4G02090.1* and *Asa2G00237.1/Asa2G00240.1* showed distinct expression response to GA3 treatment (Figure 6; Supplementary Table S3). Interestingly, the expression of these three genes in response to GA3 treatment was observed during the transition from dark to light, but not in constant light or dark conditions. Furthermore, the associated transcriptomic analysis revealed a significant correlation between the transcript abundance of DELLA-like genes, *Asa4G02090.1*, and bulb weight and diameter (*p = 0.003784* and *0.001473*, respectively). Also, the expression of *Asa2G00237.1/Asa2G00240.1* displayed a significant association with the clove number trait in 102 garlic accessions (*p = 0.03147*; Figure 7), indicating a role of these three DELLA-like genes in bulb growth. Collectively, our results indicate that GA3 could result in varied expression of DELLA-like *Asa4G02090.1* and *Asa2G00237.1/Asa2G00240.1*, thereby influencing bulb growth.

## Discussion

### Gras Family in Garlic

The garlic genome is one of the most complex genomes with a large size (16.9 Gb), high heterozygosity (1.69%), and a high ratio of repetitive sequences (91.3%). Recently, the assembly of garlic genome was completed using a repertoire of five advanced sequencing methodologies (Sun et al., 2020), which made it feasible to identify the genes important for garlic growth and development in *Allium* crops. Consequently, several gene families, such as GH19, PR, and KNOX, have been systemically characterized (Anisimova et al., 2021; Filyushin et al., 2021; Zhang et al., 2021). The GRAS family is one of the most important transcription factor families that participate widely in the plant growth regulation and development. However, GRAS genes were not identified in garlic so far. In the present study, we identified 46 GRAS members in the garlic genome. The GRAS members were slight fewer than in rice (57; Tian et al., 2004) and maize (86; Guo et al., 2017), indicating a slight contraction of this gene family in garlic genome. According to the difference of grouping criterion, the number of subfamily varied across previous studies, such as eight groups identified in Arabidopsis, rice, and maize (Tian et al., 2004; Guo et al., 2017), and 17 in the report of Cenci and Rouard (2017). To obtain a fine classification, this study analyzed the distribution of garlic GRAS members in 17 reported subfamilies and revealed that 16 of these 17 groups had garlic members. Furthermore, we systematically characterized these GRAS regulators by studying their sequence alignment, gene structure, expression, chromosomal distribution, protein domains encoded by them and their respective interactions, and subcellular localizations. The identification and characterization of AsGRAS members provide an important basis for further investigation of their function in the future.

### Response of DELLAs to GA During Garlic Bulb Formation

The garlic bulb is composed of several cloves that are essentially buds in morphology. Previous studies have shown that GAs play a key role in bud formation (Zhang et al., 2020) and the DELLA protein is an essential repressor of gibberellin signal transduction (Ikeda et al., 2001; Sun and Gubler, 2004; Park et al., 2013). For example, SLR1 is the DELLA protein of rice which promotes bud outgrowth by inhibiting the degradation of MOC1 protein, leading to an increase in tiller number (Liao et al., 2019). Similarly, StGA20 induces tuber formation by reducing gibberellin activity in potatoes (Carrera et al., 2000). In addition, exogenous application of GA3 could increase tillers in Welsh onion (Yamazaki et al., 2015) and clove numbers in garlic (Liu et al., 2019). Therefore, gibberellin has a potential role in the development of garlic bulbs. However, the mechanism that gibberellin regulates bulb formation remains unclear. In the present study, we identified three *DELLA* genes, *Asa4G02090.1* and *Asa2G00237.1/Asa2G00240.1*, whose expression was observed in response to exogenous GA3 treatment and was associated with bulb traits in 102 garlic accessions. These results indicate the possible role of these three DELLA-like genes in the bulb growth. This observation provides an important evidence to explain a previous finding that spraying exogenous GAs could increase the clove number in garlic (Liu et al., 2019).

### Response of DELLAs to Light Treatment During Garlic Bulb Formation

Light and gibberellins (GAs) are two essential signals that trigger plant developmental processes and their signaling pathways show great overlap (Feng et al., 2008). Light signals can promote the accumulation of DELLA proteins by reducing the GA levels (Achard et al., 2007). In Arabidopsis and potato, the expression of GA 20-oxidase responds to photoperiod to regulate the GA biosynthesis *in vivo* (Carrera et al., 2000; Porri et al., 2014). PIL5 is a light-labile bHLH TF that interacts with phytochrome, directly binds to the promoter of DELLA, and increases the expression of the DELLA gene in the dark (Oh et al., 2007). In garlic, long daylight can promote bulb enlargement (Wu et al., 2016). GA3 treatment dramatically stimulates lateral bud formation but inhibits the growth of garlic plants and bulbs (Liu et al., 2019). Probably, enlarged size and growth of garlic bulbs are regulated by gibberellin signaling and photoperiod. However, the combined underlying mechanism of gibberellin and daylight in garlic bulb development remains poorly understood. In this study, although none of garlic DELLA-like genes showed an expression response to light treatment, the GA3 response of three DELLA-like genes were promoted in the dark environment, indicating that a potential cross-talk between the signals of GA3 and light. These findings provide insights into the response and adaptation of garlic crops to environmental changes.

## Conclusion

A total of 46 garlic GRAS genes were identified and phylogenetically divided into 16 subfamilies. There were five members in the DELLA family, three of which showed a response to exogenous GA3 treatment, with a significant association between their transcription abundances and bulb traits in 102 garlic accessions. Therefore, these three DELLA members have been proposed to be associated with bulb growth. These findings provide a valuable foundation for further studies on the functions of GRAS members in growth and development, especially bulb growth.

## Data Availability Statement

The original contributions presented in the study are included in the article/Supplementary Material, further inquiries can be directed to the corresponding authors.

## Author Contributions

TL coordinated the project, conceived and designed experiments, and corrected the manuscript. XZ performed experiments and wrote the manuscript. XY revised the manuscript. QH and YW contributed to data analysis and managed reagents. XY and GL contributed with valuable discussions. All authors contributed to the article and approved the submitted version.

## Funding

This research was financially supported by the National Agricultural Science and Technology Innovation Program of China (CAAS-ASTIP-IBFC) and Central Public-interest Scientific Institution Basal Research Fund (1610242021002).

## Conflict of Interest

The authors declare that the research was conducted in the absence of any commercial or financial relationships that could be construed as a potential conflict of interest.

## Publisher’s Note

All claims expressed in this article are solely those of the authors and do not necessarily represent those of their affiliated organizations, or those of the publisher, the editors and the reviewers. Any product that may be evaluated in this article, or claim that may be made by its manufacturer, is not guaranteed or endorsed by the publisher.

## Footnotes

^1^ www.arabidopsis.org/index.jsp

^2^ https://www.ncbi.nlm.nih.gov/Structure/bwrpsb/bwrpsb.cgi

^3^ http://meme-suite.org/tools/meme

^4^ http://web.expasy.org/protparam/
