# Supplementary material for: Genome-wide Identification and Characterization of the GRAS Transcription Factors in Garlic (Allium sativum L.)
Source: Front Plant Sci. 2022 Apr 12;13:890052. doi: 10.3389/fpls.2022.890052 (PMC9039536; doi:10.3389/fpls.2022.890052)
Supplement: Supplementary file 1 [file Presentation_1.PDF]

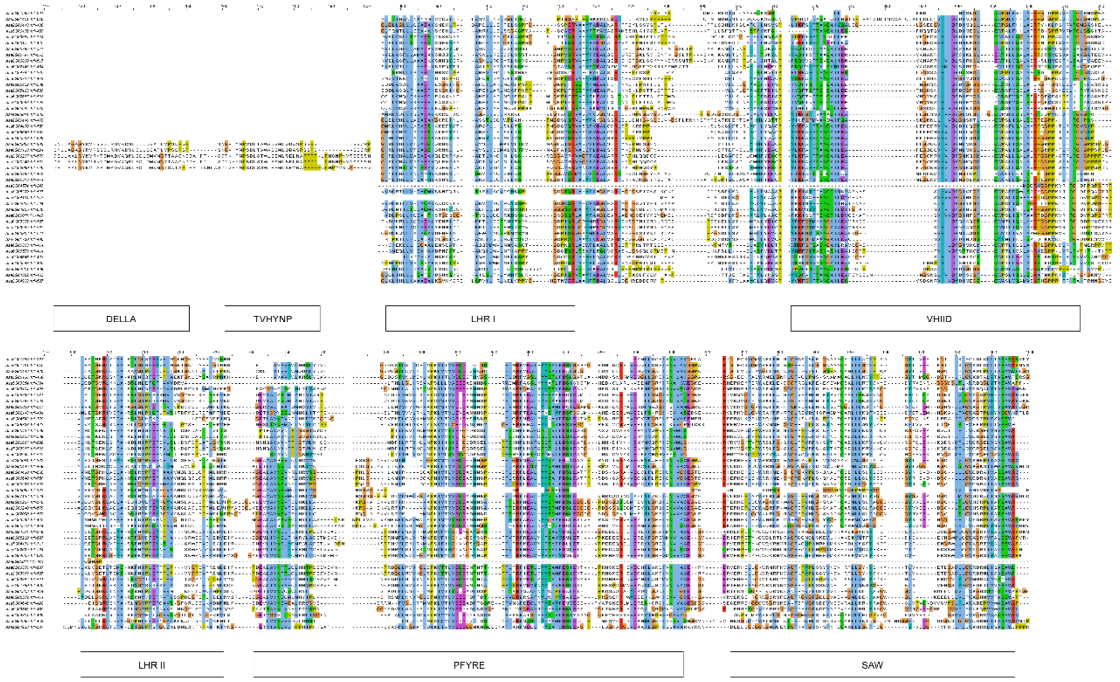

Supplementary Figure S1 | Multiple sequence alignment of the 46 AsGRAS protein family. The locations and boundaries of the five specific conserved regions LHRI, VHIID, LHR II, PFYRE, and SAW are presented within the GRAS domains. Two specific conserved regions DELLA and TVHYNP are presented within the DELLA domain of the 5 AsDELLA proteins.

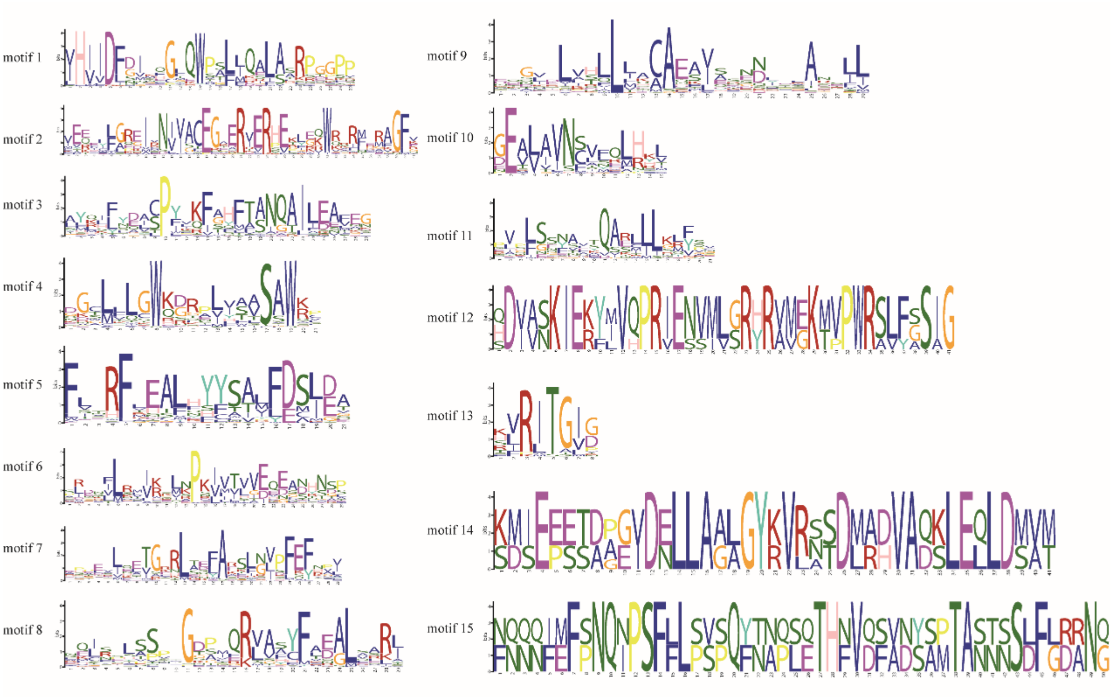

Supplementary Figure S2 | Amino acid sequences of each motif. The font size represents the frequency of the respective amino acid.
